# Chronic tramadol abuse as a cause of serotonin syndrome

**DOI:** 10.1016/j.toxrep.2026.102237

**Published:** 2026-03-09

**Authors:** Robert L.A. van Gemert, Luuk Wansink, Femke M.J. Gresnigt

**Affiliations:** a Flevoziekenhuis, Hospitaalweg 1, Almere 1315 RA, Netherlands; b OLVG, Oosterpark 9, Amsterdam 1091 AC, Netherlands; c Nationaal Vergiftigingen Informatie Centrum, UMC Utrecht, Utrecht, Netherlands; d Meander Medisch Centrum, Maatweg 3, Amersfoort 3813 TZ, Netherlands

**Keywords:** Tramadol, Serotonin syndrome, Intoxication, Serotonergic drugs, Chronic abuse

## Abstract

Serotonin syndrome is characterized by autonomic hyperactivity, altered mental status, and neuromuscular abnormalities, ranging from mild to life-threatening manifestations. Tramadol has been associated with serotonin syndrome, predominantly in combination with other serotonergic agents or following acute overdose. Reports describing serotonin syndrome caused by chronic high-dose tramadol use as a single serotonergic agent are scarce. A case of serotonin syndrome in a young adult with chronic high-dose tramadol dependence in the absence of co-ingested serotonergic drugs is presented. A 22-year-old man with tramadol dependence (800 mg daily) presented to the emergency department with anxiety and agitation. Physical examination revealed diaphoresis, mydriasis, tachycardia (130 beats/min), hypertension (140/100 mmHg), hyperreflexia, and inducible ankle clonus. Laboratory investigations, head computed tomography, and urine toxicology screening were unremarkable. Based on the presence of inducible clonus and hyperreflexia in the setting of serotonergic exposure, the Hunter Serotonin Toxicity Criteria were fulfilled. Tramadol was discontinued and replaced with methadone. The patient recovered completely within 48 h. This case illustrates that chronic high-dose tramadol use alone may precipitate serotonin syndrome. Clinicians should remain alert to this potentially life-threatening complication, even in the absence of concomitant serotonergic medication.

## Highlights

• Serotonin syndrome can result from chronic high-dose tramadol use.
• Patient met Hunter Serotonin Toxicity Criteria for diagnosis.
• Clinicians should monitor for serotonin syndrome in tramadol abuse.

Serotonin syndrome is a serious drug-induced syndrome attributable to increased serotonin concentrations in the peripheral and central nervous system [1]. Clinically, it is characterized by a triad of autonomic instability, altered mental status and neuromuscular hyperactivity [1]. The diagnosis is clinical and is commonly established using the Hunter Serotonin Toxicity Criteria. Serotonin syndrome is commonly associated with the concomitant use (or abuse) of serotonergic drugs, primarily selective serotonin reuptake inhibitors, monoamine oxidase inhibitors, or tricyclic antidepressants [1]. Tramadol, a centrally acting analgesic, exerts its effect through partial μ-opioid receptor agonism and inhibition of norepinephrine and serotonin reuptake [2]. Evidence describing serotonin syndrome resulting from chronic high-dose tramadol use without co-ingestion of other serotonergic drugs remains limited. Ali et al. [3] reported that among 71 patients with tramadol toxicity, 50 (70%) reported tramadol abuse, and 29 (41%) were diagnosed with serotonin syndrome. A prospective, observational case series by Tashakori et al. [4] showed that out of 158 patients with tramadol intoxication, of whom 90 (57%) with tramadol only and 6 (4%) with tramadol dependency, 8 (5%) received treatment for mild serotonin syndrome. However, detailed descriptions of serotonin syndrome attributable solely to chronic high-dose tramadol use are scarce. We present a patient who developed serotonin syndrome caused by chronic high-dose tramadol abuse.

A 22-year old male was brought to the emergency department by ambulance due to anxious behavior. His medical history included depression and tramadol dependence, prescribed off-label by the general practitioner for antidepressant-resistant depression, 400 milligrams, twice daily. Five months prior, he had attempted suicide by ingesting a tramadol overdose. Since then, his mother managed his tramadol dosages.

Upon examination, he exhibited anxious behavior, diaphoresis, and mydriasis. Vital signs were as follows: blood pressure 140/100 mmHg, heart rate 130 beats per minute, respiratory rate 13 per minute, and body temperature 36.7 °C. Neurological examination revealed hyperreflexia, particularly in the lower extremities, and an inducible ankle clonus. Laboratory results (Table 1) were within normal limits. A head computed tomography scan showed no abnormalities. Urine toxicology screening (*Quidel Triage® Drug Screen Panel 94600*) was negative for recreational drugs. The patient was admitted, with an abrupt cessation of tramadol, which was substituted with methadone. The patient’s symptoms resolved within 48 h, and he was discharged in stable condition.

**Table 1:** Laboratory results.

**Parameter**	**Result**	**Reference range**	**Unit**
*Hemoglobin*	9.0	8.5–11	mmol/L
*Hematocrit*	0.45	0.4–0.5	L/L
*Mean Corpuscular Volume*	88	80–100	fL
*Erythrocytes*	4.8	4.5–5.5	10^12/L
*Leukocytes*	5.5	4.0–10.0	*10^9/L
*Thrombocytes*	250	150–400	*10^9/L
*C-reactive protein*	< 5	< 5	mg/L
*e* Glomerular Filtration Rate *(CKD-EPI)*	90	> 60	mL/min/1.73 m^2
*Urea*	6.0	2.0–8.0	mmol/L
*Creatinine*	62	53–97	μmol/L
*Potassium*	4.0	3.4–4.9	mmol/L
*Sodium*	139	134–145	mmol/L

Simultaneous ingestion of other serotonergic agents or an acute tramadol overdose was unlikely, as the patient denied this and his mother observed him the preceding 24 h, confirmed his prescribed tramadol dose, and was not missing any tablets. However, plasma tramadol concentrations were not measured, which constitutes a limitation of this report. Physicians should remain vigilant regarding the potential for serotonin syndrome in patients with chronic tramadol abuse, particularly when prescribing other serotonergic drugs to these patients.

## CRediT authorship contribution statement

**Luuk Wansink:** Writing – review & editing, Conceptualization. **gresnigt Femke:** Writing – review & editing, Validation, Supervision, Methodology, Conceptualization. **van Gemert Robert Louis Arnoldus:** Writing – original draft, Validation, Project administration, Methodology, Investigation.

## Funding information

This research did not receive any specific grant from funding agencies in the public, commercial, or not-for-profit sectors.

## Disclosure statement

The authors report there are no competing interests to declare.

## Declaration of Competing Interest

The authors declare that they have no known competing financial interests or personal relationships that could have appeared to influence the work reported in this paper.

## Data Availability

Data will be made available on request.
